# Drug Retention Rates of Janus Kinase Inhibitors in Rheumatoid Arthritis Patients with Therapy-Induced Lymphopenia

**DOI:** 10.3390/jcm12144827

**Published:** 2023-07-21

**Authors:** Jumpei Temmoku, Masayuki Miyata, Eiji Suzuki, Yuya Sumichika, Kenji Saito, Shuhei Yoshida, Haruki Matsumoto, Yuya Fujita, Naoki Matsuoka, Tomoyuki Asano, Shuzo Sato, Hiroshi Watanabe, Kiyoshi Migita

**Affiliations:** 1 Department of Rheumatology, Fukushima Medical University School of Medicine, 1 Hikarigaoka, Fukushima 960-1295, Fukushima, Japan; temmoku@fmu.ac.jp (J.T.); ysumiti@fmu.ac.jp (Y.S.); s3xbck2p@fmu.ac.jp (K.S.); shuhei-y@fmu.ac.jp (S.Y.); haruki91@fmu.ac.jp (H.M.); fujita31@fmu.ac.jp (Y.F.); naoki-11@fmu.ac.jp (N.M.); asanovic@fmu.ac.jp (T.A.); shuzo@fmu.ac.jp (S.S.); h-watanabe@kawamata.saiseikai.or.jp (H.W.); 2 Department of Rheumatology, Japanese Red Cross Fukushima Hospital, Yashima 7-7, Fukushima 963-8558, Fukushima, Japan; fukuintyoumm@fukushima-med-jrc.jp; 3 Department of Rheumatology, Ohta-Nishinouchi Hospital, 2-5-20 Nishinouchi, Koriyama 963-8558, Fukushima, Japan; azsuzuki@ohta-hp.or.jp

**Keywords:** rheumatoid arthritis, Janus kinase inhibitor, lymphopenia, drug retention rate

## Abstract

Objectives: To determine whether drug-induced lymphocytopenia is associated with drug retention rates of JAKi (tofacitinib or baricitinib) in rheumatoid arthritis (RA) patients. Methods: Patients with RA who were initiated with tofacitinib (*n* = 38) or baricitinib (*n* = 74) between July 2015 and July 2022 and continued for at least 4 months were enrolled in this study. Absolute lymphocyte count (ALC) value was obtained pre-treatment and monthly after initiation of JAKi (up to 4 months). Associations between ALC nadir at an early phase (up to 4 months) from JAKi initiation and drug retention rates were analysed. Results: 112 patients (87 females; age, 71.2 ± 14.0 years; disease duration, 9.2 ± 10.5 months; DAS28-CRP, 3.60 ± 1.12; DAS28-ESR, 4.43 ± 1.29; CDAI, 17.9 ± 12.9; C-reactive protein, 3.07 ± 3.43 mg/dL; and lymphocyte count, 1361.9 ± 538.7 per μL) treated with tofacitinib or baricitinib were retrospectively analysed. Lymphocytopenia (>10% decline in lymphocyte count to pre-treatment basal levels) was observed in a quarter of RA patients treated with JAKi (tofacitinib; 16 baricitinib; 14). RA patients with lymphopenia were associated with the lower drug retention rates of tofacitinib compared to those without lymphocytopenia. The reduced drug retention rates in patients with lymphocytopenia were attributed to the discontinuation of tofacitinib due to AEs. Whereas lymphocytopenia was not associated with lower drug retention rates of baricitinib. Pre-treatment absolute lymphocyte counts did not affect the drug retention rates of JAKi in patients with RA. Conclusions: These findings suggest that lymphopenia during the first 4 months from the initiation of JAKi is associated with reduced drug retention rates in patients with RA due to AEs, which is exclusively associated with the use of tofacitinib.

## 1. Introduction

The Janus kinase (JAK)-signal transducer and activator of transcription (STAT) pathway is involved in the signal transduction of cytokine receptors [1], and inhibition of JAKs appears to be a promising strategy in immune-mediated diseases [2]. To date, various JAK inhibitors (JAKi) have been approved for the treatment of rheumatoid arthritis (RA) and autoimmune diseases [3]. Their efficacy and safety profile have been shown to be comparable to those of biologic disease-modifying antirheumatic drugs (bDMARDs) [4]. However, in addition to its antirheumatic effect, recent studies have focused on adverse events (AEs) [5]. As with bDMARDs, serious infections have been observed in RA patients treated with JAKi [6]. Haematological disorders were observed following JAKi treatment [7]. Lymphopenia is of particular clinical relevance because of its association with an increased number of AEs, including infections [8]. However, few reports have examined the real-world treatment outcomes or safety of JAKi in RA patients in relation to haematological abnormalities, including lymphocytopenia in RA patients. It is difficult to apply the therapeutic evidence obtained from clinical trials to the real world because the increase in comorbidities and treatment-related risks may not be comparable to those of real-world RA patients [9].

In general, immune dysfunction, including lymphocytopenia, is associated with the pathogenesis of some cancers, inflammation, and infectious diseases [10]. Furthermore, recent studies have demonstrated that lymphocytopenia reduces longevity in the general population [11]. Whether drug-induced lymphopenia is associated with clinical outcomes in patients with RA has not been well studied. This study aimed to investigate drug retention rates according to trends in lymphocyte counts after initiation of JAK inhibitors in patients with RA in a real-world setting. We also investigated whether lymphocyte counts reflect the rates of JAKi discontinuation due to infectiveness or AEs in RA patients treated with tofacitinib or baricitinib using an observational retrospective cohort study.

## 2. Materials and Methods

### 2.1. Patients and Study Design

We conducted a multicenter retrospective cohort study to evaluate the retention rate of JAKi and factors affecting the discontinuation of JAKi in patients with RA. Our cohort consisted of patients treated at the department of rheumatology in Fukushima Medical University Hospital, Japanese Red Cross Fukushima Hospital and Ohta Nishinouchi Hospital. Between June 2015 and July 2022, 184 patients with RA who were initiated with JAKi were enrolled in this study. All the patients were diagnosed with RA according to the 2010 ACR/European League Against Rheumatism classification criteria for RA [12]. The study was approved by the Institutional Review Boards of Fukushima Medical University (No. 2019-097), Japanese Red Cross Fukushima Hospital (No. 55) and Ohta Nishinouchi Hospital (No. 2022–8).

### 2.2. Clinical Evaluations

At the start of JAKi treatment, baseline data were collected from medical records, including demographics, clinical data (disease duration, presence of the anti-citrullinated protein/peptide antibody [ACPA]), evaluation of disease activity (swollen joint count, tender joint count, patient global assessment, and physician global assessment), C-reactive protein [CRP], and haematological data. The treatment was at the discretion of the attending physician based on the clinical conditions and the patient’s intentions.

### 2.3. Drug Retention Rates

Drug retention rates were retrospectively evaluated at monthly intervals until definitive treatment was interrupted. If the treatment was discontinued, the reasons for discontinuation were recorded. Decisions to discontinue JAKi were carefully determined by highly experienced rheumatologists based on the evaluation of clinical findings, laboratory data, and radiological examinations. The reasons for discontinuation were classified into two major categories: (1) lack of effectiveness (including primary and secondary); and (2) adverse events including infections, malignancy and major adverse cardiovascular events (MACE).

### 2.4. Statistical Analysis

Continuous variables are shown as mean ± standard deviation, and categorical variables are expressed as frequencies (percentages). Chi-squared test was used for comparing qualitative variables, and the Mann–Whitney’s U test was used for comparing continuous variables, as appropriate. Drug retention rate was analysed using Kaplan–Meier plots and assessed using the log-rank test. Statistical analyses were performed using SPSS Statistics software (version 25.0; IBM Corp., Armonk, NY, USA). Two-tailed *p* values < 0.05 were considered indicative of statistical significance.

## 3. Results

### 3.1. Study Population

This study retrospectively analysed 136 RA patients who were initiated with tofacitinib or baricitinib between June 2015 and July 2022. Of these patients, 112 patients who continued on tofacitinib or baricitinib for at least 4 months were enrolled in the study. Demographic and disease-related characteristics of the patients are shown in Table 1. Among these patients, 38 patients were treated with tofacitinib and 74 patients were treated with baricitinib. The majority (77.7%, 87/112) were women, with a mean age of 71.2 ± 14.0 years at cohort entry. Mean disease duration was 9.2 ± 10.5 years and ACPA positivity was 66.4% (71/107). The mean of the DAS28-CRP, DAS28-ESR and CDAI were 3.60 ± 1.12, 4.43 ± 1.29 and 17.9 ± 12.9, respectively. The mean dose of the concomitant medications were glucocorticoid (GC; 3.02 ± 23.7 mg/day (20.5%) and MTX was 6.65 ± 2.07 mg/week (41.3%). Forty-three (38.4%) patients had a history of bDMARD use. Mean follow-up periods after initiation of JAKi was 27.4 ± 15.8 months. In addition, a comparison of clinical characteristics for RA patients treated with baricitinib and tofacitinib are shown in Table 2. There was no significant difference of clinical characteristics including age and gender between the baricitinib and tofacitinib groups.

### 3.2. Changes in Lymphocyte Subsets

**Figure 1:**
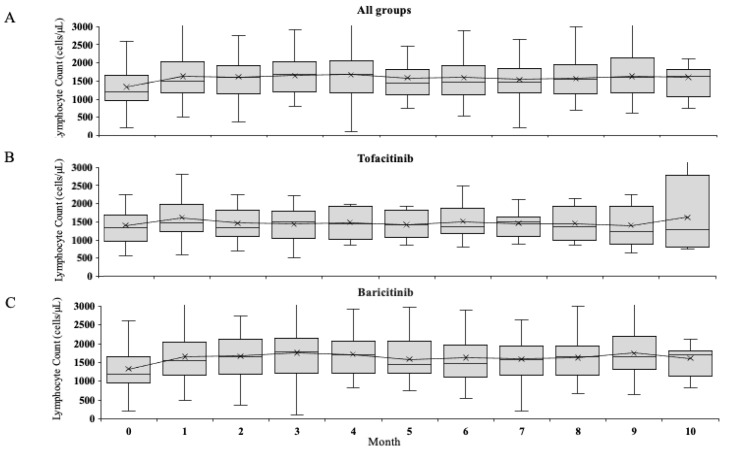
Median absolute leukocyte counts over time in RA patients initiated with JAKi. The changes of absolute leukocyte counts pre-treatment and over time with treatments in RA patients initiated with JAKi ((**A**); Total, (**B**); tofacitinib, (**C**); baricitinib). Values are the median and 25th–75th percentiles. ALC was increased at 1 month to 4 months compared with the pre-treatment time point, however, there was no statistical differences at each point (1–4 months).

We analysed the changes in the absolute lymphocyte count (ALC) during the early phase (0–4 months) from the initiation of JAKi among the RA patients initiated with JAKi. ALC were marginally increased at one month from the initiation of JAKi, however, there was no statistically significant difference compared to those of the pre-treatment phase (Figure 1). These tendencies were observed in both tofacitinib and baricitinib-treated patients and the increased lymphocyte counts seem to be returned within the base line at 3 months in tofacitinib and at 6 months in baricitinib-treated patients. Transient lymphopenia (>10% decline in lymphocyte count to pre-treatment basal levels) was observed in a quarter of RA patients treated with JAKi (tofacitinib; 16/38 baricitinib; 14/74). There was no difference in the incidence of lymphopenia between the very elderly group (75 ≤ age) and the non-very elderly group (age < 75) (27.7% vs. 26.2%).

### 3.3. Drug Retention Rates and Reasons for Discontinuation

Among 112 patients initiated with JAKi, tofacitinib was discontinued in 10 patients (26.3%) due to lack of effectiveness and in 13 patients (34.2%) due to AEs. Baricitinb was discontinued in 15 patient (20.3%) due to lack of effectiveness and in nine patients (12.2%) due to AEs. Any cases of JAKi discontinuation due to patient adherence or lymphopenia were not observed. Details of the adverse events leading to drug discontinuation are shown in Table 3. In regard to tofacitinib, AEs included infection, six; malignancy, five; liver impairment, one; and cardiovascular complications; one. In regard to baricitinb, AEs included infection, four; malignancy, three; liver impairment, one; and exacerbation of interstitial pneumonia, one. As shown in Table 4, lymphopenia was observed more frequently in RA patients treated with tofacitinib (16/38, 42.1%) than in RA patients treated with baricitinib (14/78, 17.9%). Discontinuation of the drug due to lack of effectiveness was the most common in baricitinib-treated patients with lymphopenia, while adverse events including infection were the most common in tofacitinib-treated patients with lymphopenia.

### 3.4. Drug Retention Rates between RA Patients with or without Lymphocytopenia

We compared the demographic data between RA patients with or without lymphocytopenia after the initiation of JAKi; however, there was no significant difference in all variables including age and gender (Table 5).

**Figure 2:**
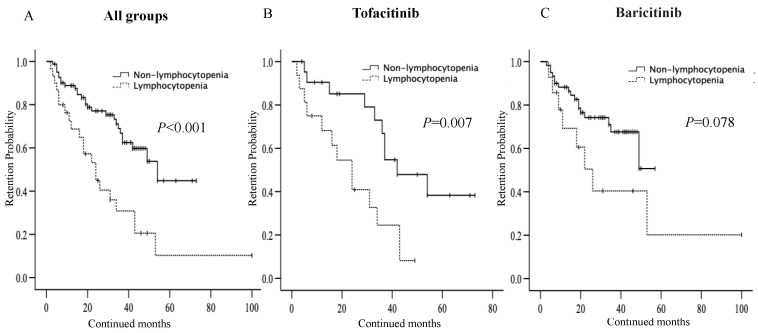
(**A**): Kaplan–Meier curve related to overall drug retention rates of JAKi between RA patients with and without lymphocytopenia (>10% decline in lymphocyte counts to basal levels). (**B**): Kaplan–Meier curve related to overall drug retention rates of tofacitinib between RA patients with and without lymphocytopenia. (**C**): Kaplan–Meier curve related to overall drug retention rates of baricitinib between RA patients with and without lymphocytopenia. Overall drug retention rates of tofacitinib were significantly lower in RA patients with lymphocytopenia. Whereas there was no significant difference in overall drug retention rates of baricitinib between RA patients with and without lymphocytopenia.

**Figure 3:**
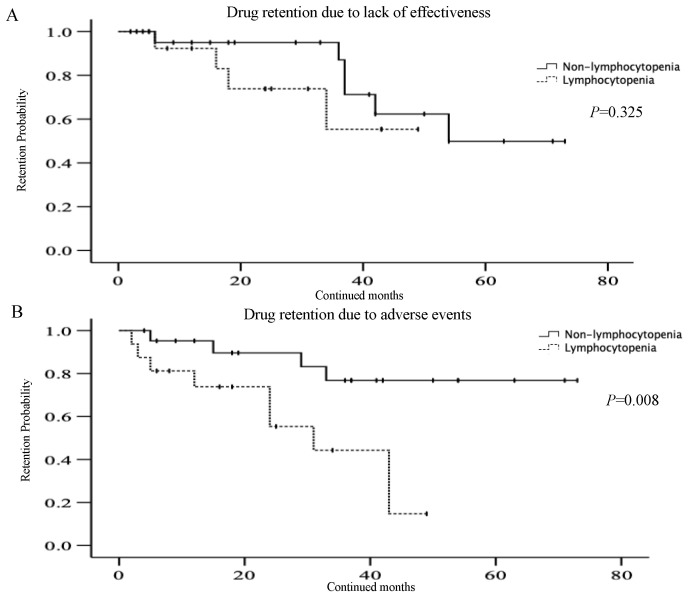
(**A**): Kaplan–Meier curve of drug retention rates of tofacitinib according to the discontinuation due to lack of effectiveness between RA patients with and without lymphocytopenia. (**B**): Kaplan–Meier curve of drug retention rates of tofacitinib according to the discontinuation due to AEs between RA patients with and without lymphocytopenia. Drug retention rates of tofacitinib according to the discontinuation due to AEs were significantly lower in RA patients with lymphocytopenia compared to those without lymphocytopenia.

We also compared the overall drug retention rates between RA patients with or without lymphocytopenia (>10% decline in lymphocyte counts to basal levels) observed in the early phase of JAKi initiation using the Kaplan–Meier curves. The overall drug retention rate of JAKi was significantly lower in RA patients with lymphocytopenia compared to those without lymphocytopenia (Figure 2A). Lymphocytopenia was differentially associated with the lower drug retention rates according to the type of JAKi. Patients with lymphocytopenia were associated with lower drug retention rates in the tofacitinib-treated group (Figure 2B). Whereas lymphocytopenia was not associated with lower drug retention rates in the baricitinib-treated group (Figure 2C). Furthermore, the reduced drug retention rates were caused by the discontinuation due to AEs, not to a lack of effectiveness in tofacitinib-treated patients (Figure 3). In contrast, there was no significant difference in the drug retention rates according to the discontinuation due to AEs between RA patients with and without lymphocytopenia in baricitinib-treated patients.

**Figure 4:**
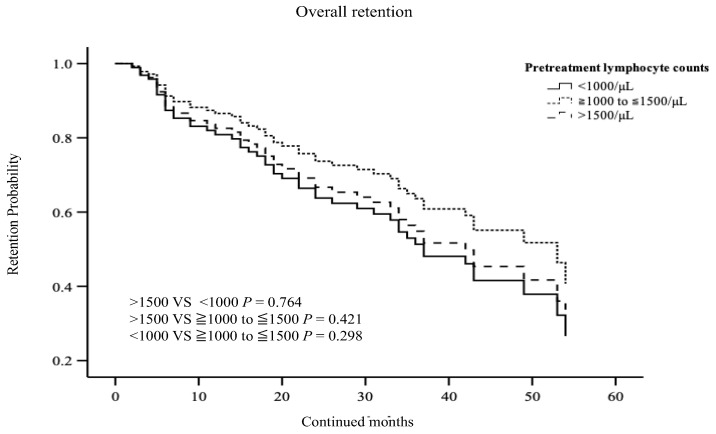
Kaplan–Meier curve related to drug retention rate of JAKi among RA patients stratified according to the pre-treatment absolute leukocyte count. Mild lymphocytopenia (>1000/μL to 1500/μL) or severe lymphocytopenia (<1000/μL) and without lymphopenia (>1500/μL), respectively. The overall drug retention rates of JAKi were not affected by the pre-treatment absolute leukocyte count.

Finally, we compared the overall drug retention according to the pre-treatment lymphocyte counts among the patients with RA using the Kaplan–Meier curves. However, pre-treatment lymphocyte counts did not affect the drug retention rates of JAKi in patients with RA (Figure 4).

## 4. Discussion

Most cytokines and growth factors that bind to their receptors result in phosphorylation of receptor-associated JAKs [13]. JAKi selectively interfere with the adenosine triphosphate-binding site of JAKs, resulting in suppression of downstream signalling pathways [14]. Considering the role of cytokines and growth factors in immune cell survival and activation, the suppressive effects of JAKi on these cells are presumed to silence the autoimmune state [2]. The JAK-STAT signalling pathway is downstream of certain cytokine receptors that are known to be involved in RA pathogenesis [15]. Janus kinase inhibitors are promising for treating RA and several other inflammatory conditions [3]. Some AEs associated with JAKi are predicted by mechanisms related to the blockade of cytokines, which could explain the risk of various infections [16]. Recently, JAKi have been reported to be associated with potentially serious effects, including malignancy and major adverse cardiovascular events (MACE) [17]. Thus, the latest EULAR recommendations state that risk factors including age over 65 years, hypertension, and history of malignancy should be evaluated when using JAKis inhibitors [18].

In this study, we evaluated the changes of lymphocyte counts in patients with RA (n = 112) initiated with JAKi and assessed whether lymphocyte changes are associated with the drug discontinuation, including the effectiveness or safety of JAKi. We provided information on the changes in total lymphocyte counts and their effect on the clinical course of patients with JAKi-initiated RA. In our date, absolute lymphocyte counts were marginally increased after the initiation of JAKi, however, there was no significant difference compared to those of pre-treatment baseline phase in patients with RA. However, one-fourth of the patients initiated with JAKi showed a transient decline in lymphocytes (>10% reduction to basal levels) in the early phase (up to 4 months) from the initiation of JAKi. These RA patients with lymphopenia presented with significantly lower drug retention rates of JAKi compared to those without lymphocytopenia in tofacitinib-treated patients. Furthermore, the reduced drug retention rates were caused by the discontinuation of JAKi due to AEs in tofacitinib-treated patients. Conversely, in baricitinib-treated patients, lymphocytopenia was not associated with the lower drug retention rates of baricitinib.

It is possible that each JAKi affects different lymphocyte subsets, which is related to the differential treatment outcomes, including AEs. However, the changes in each lymphocyte subset were not analysed during the course of treatment with JAKi, therefore, our results did not indicate the mechanism by which lymphocytopenia influenced the drug retention rates of JAKi in RA patients. Previous studies have demonstrated that treatment of RA patients with tofacitinib resulted in a reduction of peripheral CD4^+^ T lymphocytes, and such a reduction did not correlate with improvement of disease activity [19]. However, a low number of CD8^+^ T cells predicts infectious AEs in patients with RA treated with tofacitinib [20]. Analysis of lymphocyte subsets during the course of baricitinib treatment showed preferential loss of Th1 cells compared to Th17 cells and preserved Treg counts, suggesting that Th1 cells are inhibited by baricitinib treatment [21]. It was also demonstrated that lymphopenia was associated with a slightly higher rate of overall infection among patients treated with baricitinib, but serious infections were uncommon in patients with lymphopenia in baricitinib-treated patients [22]. Taken together, it is possible that there is a correlation between tofacitinib-induced lymphopenia and its discontinuation due to AEs by affecting the particular immune phenotypic cells. Our findings suggest that tofacitinib-induced lymphocytopenia could be related to the reduced drug retention rates due to AEs by influencing the immune system. Although tofacitinib was originally designed to be a JAK3-selective inhibitor, however, it is now considered to be a pan-JAK inhibitor. In contrast, baricitinib is a potent JAK1/2 inhibitor [23]. In clinical data, lymphocytopenia (<500 cells/mL) occurred in 8.3/100 patient years in tofacitinib-treated patients, however, lymphocytopenia was uncommon (<1% of patients) in baricitinib-treated patients [23]. Jak3-deficient mice had severe B cell and T cell lymphopenia [24]. Taken together, JAK3 inhibition may be related to the JAKi-induced lymphocytopenia.

Lymphocytopenia in the general population is associated with reduced longevity independent of age or traditional risk factors [11]. ALC seems to be associate with cardiovascular disease (CVD) and RA disease activity suggesting the linkage between rheumatoid inflammation and lymphopenia [25]. Lymphocytopenia, disease activity, and comorbidity burden were shown to be independent risk factors for infection in RA [26]. Rheumatoid arthritis is associated with inherent immune dysfunction, and JAKi can affect lymphocyte counts by reducing receptor signaling. Our data suggest that lymphocytopenia during the early phase after JAKi initiation could be predictive of drug discontinuation of a particular JAKi due to AEs. However, whether these effects of JAKi are mediated directly via JAK-driven signalling pathways on lymphocyte or depend on the selectivity of JAK inhibitions are unclear. Additional studies are required to characterize the lymphocyte homeostasis and its relationship with clinical course in JAKi-treated RA patients.

Our study has several limitations. First, given its retrospective design, the study was subjected to several possible biases. Second, the choice of treatment and decision to discontinue treatment were made at the discretion of each rheumatologist with no standardized protocol. Third, the sample size was relatively small. Fourth, the JAKi used in this study were limited to tofacitinib and baricitinib. Finally, we select lymphocytopenia at the limited phase (up to 4 months from the initiation of JAKi) as a variable due to the nature of cohort studies with the increasing terminated cases, with inherent biases.

## 5. Conclusions

Our study demonstrated that the drug retention rates of tofacitinib were affected by the occurrence of lymphocytopenia at the early phase of tofacitinib initiation in patients with RA. These findings suggest that tofacitinib-induced lymphopenia may predict the risk of AEs in RA patients. However, the results of this study were from a small retrospective study, and further investigation is required to confirm our findings.

## Figures and Tables

**Table 1:** Baseline clinical characteristics in RA patients treated with JAKi.

Characteristic	*n* = 112
Age (years)	71.2 ± 14.0
Female, *n* (%)	87 (77.7)
JAKi use, *n*	TOF 38, BAR 74
Disease duration (years)	9.2 ± 10.5
RF-positive, *n* (%)	69/107 (64.5)
ACPA-positive, *n* (%)	71/107 (66.4)
CRP (mg/dL)	3.07 ± 3.43
MMP-3 (ng/mL)	240.0 ± 299.4
Lymphocyte count (/μL)	1361.9 ± 538.7
DAS28-CRP	3.60 ± 1.12
DAS28-ESR	4.43 ± 1.29
CDAI	17.9 ± 12.9
eGFR (mL/min)	73.1 ± 22.6
CKD (eGFR < 60), *n* (%)	26 (23.2)
Interstitial lung disease, *n* (%)	19 (16.9)
MTX use, *n* (%)	46 (41.3)
MTX dose (mg/week)	6.7 ± 2.1
GC use, *n* (%)	23 (20.5)
GC dose (mg/day)	3.0 ± 23.7
Prior bDMARDs use, *n* (%)	43 (38.4)
Follow up periods (month)	27.4 ± 15.8

Values are presented as mean ± standard deviation or percentage; RA: rheumatoid arthritis; JAKi: janus kinase inhibiter; RF: rheumatoid factor; ACPA: anti-citrullinated peptide antibody; CRP: c-reactive protein; MMP-3: matrix metalloproteinase-3; DAS28: disease activity score 28; ESR: erythrocyte sedimentation rate; CDAI: clinical disease activity index; eGFR: estimated glomerular filtration rate; CKD: chronic kidney disease; MTX: methotrexate; GC: glucocorticoid; bDMARDs: biological disease-modifying antirheumatic drugs.

**Table 2:** Comparison of clinical characteristics between RA patients treated with a baricitinib and tofacitinib.

Characteristic	Baricitinib (*n* = 74)	Tofacitinib (*n* = 38)	*p*-Value
Age (years)	70.8 ± 15.2	72.0 ± 11.3	0.943
Female, *n* (%)	59 (79.7)	28 (73.7)	0.467
Disease duration (years)	9.4 ± 11.0	9.0 ± 9.4	0.965
RF-positive, *n* (%)	45/71 (63.4)	24/36 (66.7)	0.296
ACPA-positive, *n* (%)	47/71 (66.2)	24/36 (66.7)	0.961
CRP (mg/dL)	3.0 ± 3.5	3.2 ± 3.4	0.342
DAS28-CRP	3.5 ± 1.0	3.7 ± 1.3	0.795
eGFR (mL/min)	73.4 ± 23.6	72.6 ± 20.9	0.943
Interstitial lung disease, *n* (%)	13 (17.6)	6 (15.8)	0.812
MTX use, *n* (%)	26 (35.1)	20 (52.6)	0.075
GC use, *n* (%)	15 (20.3)	8 (21.1)	0.923
Prior bDMARDs use, *n* (%)	25 (33.8)	18 (47.4)	0.162
Follow up periods (month)	24.0 ± 15.2	29.4 ± 16.9	0.110

Values are presented as mean ± standard deviation or percentage; RA: rheumatoid arthritis; RF: rheumatoid factor; ACPA: anti-citrullinated peptide antibody; CRP: c-reactive protein; DAS28: disease activity score 28; eGFR: estimated glomerular filtration rate; MTX: methotrexate; GC: glucocorticoid; bDMARDs: biological disease-modifying antirheumatic drugs.

**Table 3:** Adverse events leading to JAKi discontinuation.

Adverse Events	Total *n* = 22	Baricitinib *n* = 9	Tofacitinib *n* = 13
Infection	10	4 (Pneumonia 3, Herpes Zoster 1)	6 (Pneumonia 4, Pyothorax 1, Cholecystitis)
Malignancy	8	3 (Lymphoma 2, Lung 1)	5 (Lymphoma 2, Lung 2, Colon 1)
Liver impairment	2	1	1
Exacerbation of interstitial pneumonia	1	1	0
Cardiovascular disease	1	0	1
			(Cerebral hemorrhage)

JAKi: janus kinase inhibiter.

**Table 4:** Outcome of JAKi retention in RA patients with lymphopenia (>10%).

Lymphopenia (+)	Baricitinib *n* = 14/78 (17.9%)	Tofacitinib *n* = 16/38 (42.1%)
Discontinued of JAKi	10 (71.4%)	13 (81.3%)
Cause		
Lack of effectiveness	9 (64.3%)	4 (25.0%)
Adverse events	1 (7.1%)	9 (56.3%)
Infection	1 (7.1%)	6 (37.5%)
Malignancy	0	2 (12.5%)
Liver damage	0	1 (6.3%)
(Continued)	4 (28.6%)	3 (18.7%)

JAKi: janus kinase inhibiter, RA: rheumatoid arthritis.

**Table 5:** Comparison of characteristics between RA patients with and without lymphocytopenia.

	Lymphocytopenia (+) (*n* = 30)	Lymphocytopenia (−) (*n* = 82)	*p*-Value
Age (years)	71.6 ± 14.0	71.0 ± 14.1	0.851
Female, *n* (%)	25 (83.3)	62 (75.6)	0.385
Disease duration (years)	9.4 ± 12.2	9.2 ± 9.9	0.917
RF-positive, *n* (%)	21/29 (72.4)	48/78 (61.5)	0.296
ACPA-positive, *n* (%)	21/28 (75.0)	50/79 (63.3)	0.260
CRP (mg/dL)	3.3 ± 4.0	3.0 ± 3.2	0.713
DAS28-CRP	3.9 ± 1.1	3.5 ± 1.1	0.184
CDAI	19.0 ± 14.4	17.6 ± 12.5	0.698
eGFR (mL/min)	74.3 ± 24.7	72.7 ± 21.9	0.748
Interstitial lung disease, *n* (%)	6 (20.0)	13 (15.9)	0.605
MTX use, *n* (%)	10 (33.3)	36 (43.9)	0.314
GC use, *n* (%)	9 (30.0)	14 (17.1)	0.134
Prior bDMARDs use, *n* (%)	14 (46.7)	29 (35.4.)	0.276
Follow up periods (month)	31.2 ± 16.8	34.1 ± 16.8	0.487

Values are presented as mean ± standard deviation or percentage; RA: rheumatoid arthritis; Lymphocytopenia indicates nadir lymphocyte counts >10% reduction to basal levels; RF: rheumatoid factor; ACPA: anti-citrullinated peptide antibody; CRP: c-reactive protein; DAS28: disease activity score 28; CDAI: clinical disease activity index; eGFR: estimated glomerular filtration rate; MTX: methotrexate; GC: glucocorticoid; bDMARDs: biological disease-modifying antirheumatic drugs.

## Data Availability

The data presented in this study are available on request from the corresponding author. The data are not publicly available due to the information that could compromise the privacy of research participants.

## Abbreviations

JAKi	Janus kinase inhibitors
RA	Rheumatoid arthritis
ALC	Absolute lymphocyte count
DAS28	the disease activity score of 28 joints
CRP	C-reactive protein
ESR	erythrocyte sedimentation rate
CDAI	Clinical Disease Activity Index
AEs	adverse events
ACR	American College of Rheumatology
ACPA	anti-citrullinated protein/peptide antibody
MACE	major adverse cardiovascular events
MTX	methotrexate
bDMARD	Biological disease-modifying antirheumatic drug.
CVD	cardiovascular disease
